# QT prolongation in participants receiving bedaquiline-containing regimens: analysis of data from Phase 3 STREAM Stage 2

**DOI:** 10.5588/ijtldopen.25.0652

**Published:** 2026-05-11

**Authors:** E. Birmingham, B. Remmerie, I. Leipoldt, I. Eshun-Wilsonova, K. Callewaert, N. Bakare

**Affiliations:** 1 Johnson & Johnson, Raritan, NJ, USA;; 2 Johnson & Johnson, Beerse, Belgium;; 3 Johnson & Johnson, Durban, South Africa;; 4 Johnson & Johnson, Titusville, NJ, USA.

**Keywords:** tuberculosis, MDR-TB, QTcF, tachyarrhythmias, Torsades de Pointes

## Abstract

**BACKGROUND:**

Multidrug-resistant TB regimens are associated with higher risk of QT prolongation; understanding bedaquiline’s (BDQ) role is crucial for safety and management.

**METHODS:**

We report a pre-specified secondary analysis of data from STREAM Stage 2 (ISRCTN18148631), a Phase 3, non-inferiority trial evaluating BDQ-containing regimens in participants aged ≥15 years with multidrug/rifampicin-resistant TB. Participants were randomised to one of four regimens (two BDQ-containing and two BDQ-free). Electrocardiogram assessments were routinely conducted through Week 76, with continued monitoring for significant QT prolongation. Averaged Fridericia’s corrected QT interval (QTcF) results are reported.

**RESULTS:**

Of 588 participants, median age was 32.7 years (range 16–69) and 236 (40%) were female. QTcF gradually increased from baseline to Weeks 10–14, plateaued, then decreased during treatment. Mean QTcF change for BDQ regimens was <10 ms higher than control at all timepoints. Risk of experiencing at least one QTcF ≥500 ms was similar between control and 40-week oral BDQ regimen (hazard ratio 1.43, 95% confidence interval: 0.66–3.11).

**CONCLUSION:**

BDQ was associated with mean QTcF prolongation of <10 ms, which was not more than additive to other drugs. Rates of QTcF ≥500 ms were comparable between BDQ-containing and BDQ-free arms, indicating comparable incidences of this event within the study context.

Multidrug-resistant TB (MDR-TB) treatment regimens have significantly improved over the last decade, with better tolerability and shorter treatment durations. Updated treatment recommendations from the WHO now endorse 6- or 9-month, all-oral, bedaquiline (BDQ)-containing regimens as standard of care.^1^ However, concerns about QT interval prolongation, specifically related to BDQ, continue to persist. BDQ was granted accelerated approval in 2012 for the treatment of MDR-TB in combination therapy in the USA, and conditional approval by the European Medicines Agency in 2014.^2^ This was based on data from two Phase 2 registrational trials (TMC207-C208 and TMC207-C209), in which the safety and efficacy of BDQ administered for 24 weeks as part of a combination MDR-TB treatment regimen was examined.^3–5^ In these trials, prolongation of the Fridericia’s corrected QT (QTcF [corrected for heart rate]) interval was reported in participants receiving BDQ, though no episodes of ventricular tachyarrhythmias occurred.^3–5^ While the degree of QT prolongation is recognised as an imperfect biomarker for proarrhythmic risk,^6^ in general, there is a quantitative relationship between QT prolongation and risk of Torsades de Pointes.^7,8^ A QTcF value of >500 ms, or an increase of ≥60 ms, has been shown to be the threshold for increased risk of life-threatening arrhythmias.^7,8^ Specifically, a QT interval of >500 ms has the potential to degenerate into tachyarrhythmias.

More recently, extensive methodological, clinical, and real-world data have emerged on the use of MDR-TB drug regimens and QT interval prolongation.^9,10^ Prospective and retrospective studies report modest QTc increases, most commonly observed early in treatment, particularly when BDQ is used in combination with other QT-prolonging anti-TB agents, with a low incidence of clinically significant arrhythmias.^6,11–16^ However the individual contributions of single agents remain uncertain, as these regimens often include several drugs in combination known to be associated with QT prolongation,^17^ such as fluoroquinolones, BDQ, and clofazimine.

We present data on QT prolongation from the Phase 3 STREAM Stage 2 study (ISRCTN18148631) which evaluated two BDQ-containing regimens (a 40-week oral regimen and a 24-week regimen with 8 weeks of second-line injectable) compared to the non-BDQ control, aiming to provide a clearer understanding of BDQ’s contribution to QT prolongation when administered alongside other QT-prolonging drugs.

## METHODS

The STREAM Stage 2 trial design and methodology have been previously published.^17^ In brief, STREAM Stage 2 was a randomised, Phase 3, non-inferiority trial conducted in participants aged ≥15 years with multidrug/rifampicin-resistant TB. Participants were randomised (1:2:2:2) to one of four treatment regimens, two of which (oral and 6-month regimens) contained BDQ (Table). Participants receiving the control regimen initially received a regimen containing moxifloxacin (control-mox) until after consideration of the STREAM Stage 1 results, when levofloxacin (control-lev) replaced moxifloxacin given the higher QT prolongation safety concerns with moxifloxacin. Recruitment for the WHO regimen was terminated early due to changes in global standard-of-care MDR-TB guidelines during the trial. Similarly, and with exception of sites in India, recruitment of 6-month regimen was stopped given increased focus in the development of all-oral regimens. Electrocardiogram (ECG) analyses in this publication are restricted to participants randomised to control, oral, and 6-month regimens.

**Table.** MDR-TB regimens investigated in STREAM Stage 2.

Study arm	Regimen	Duration of regimen
A	MDR-TB regimen in accordance with 2011 WHO MDR-TB treatment guidelines.^A^	Recommended duration of 20 months
Control	MFX^B^ (or LFX), CFZ, EMB, and PZA, supplemented by injectable KM (or AM, high-dose INH, and PTO in the first 16 weeks [intensive phase^C^]).	40-week regimen, including a 16-week intensive phase
Oral	All-oral regimen of BDQ, LFX, CFZ, EMB, and PZA, supplemented by high-dose INH and PTO in the first 16 weeks (intensive phase^C^).	40-week regimen, including a 16-week intensive phase
6-month	BDQ, LFX, CFZ, and PZA supplemented by KM (injectable) and INH dose higher than doses used in regimens B and C for the first 8 weeks (intensive phase^C^).	28-week regimen, including an 8-week intensive phase

Table adapted from Goodall et al.^18^

AM = amikacin; BDQ = bedaquiline; CFZ = clofazimine; EMB = ethambutol; INH = isoniazid; KM = kanamycin; LFX = levofloxacin; MDR-TB = multidrug-resistant TB; MFX = moxifloxacin; PTO = prothionamide; PZA = pyrazinamide; WHO = World Health Organization.

A WHO-recommended regimen lasted ≥20 months. It contained PZA, a fluoroquinolone, a second-line injectable drug, ethionamide (or PTO), and either cycloserine or *P*-aminosalicylic acid for the treatment of MDR-TB, but was stopped early due to guideline changes.^1^

B MFX (regimen control-mox) was replaced by LFX (regimen control-lev) in 2018 due to the extent of QT prolongation seen in STREAM Stage 1.^19^

C The intensive phase should be extended by 4 or 8 weeks for participants whose smear has not converted.

### Participants

Eligible participants were aged ≥15 years (where approved, otherwise ≥18 years) with pulmonary TB, with evidence of resistance to at least rifampicin regardless of susceptibility to isoniazid. Participants were to be excluded from the study if they showed *Mycobacterium tuberculosis* resistance to a second-line injectable drug and/or fluoroquinolone.^18^

### ECG monitoring, data handling, and relevant definitions

Regular ECG monitoring (up to Week 76, and thereafter in participants with increased QTcF from baseline until the QTcF returned to either <10 ms increase above the baseline value, or <450 ms) with centralised and/or local review of ECGs was performed. The corrected QT interval was measured using QTcF and, if necessary, TB treatment was modified to maintain QTcF <500 ms. The treatment phase was defined as the period from randomisation to the last dose of any TB treatment + 7 days. Further details on ECG measurement are provided in the publication by Goodall et al.^18^ Of note, the analysis reported in this manuscript draws on larger ECG dataset than the Goodall et al.^18^ publication, which included central ECG data up to 76 weeks. For statistical analyses, the average of the available ECG assessments per timepoint was derived for centralised or local ECG measurements as available per timepoint. Averaged QTcF results were categorised into the following subgroups: by QTcF value (<450 ms; ≥450 to <480 ms; ≥480 to <500 ms; ≥500 ms); by change from baseline in QTcF (<30 ms; ≥30 to <60 ms; ≥60 ms). In this trial, QT prolongation was defined as ≥480 ms, while QTcF ≥500 ms, consistent with ICH (International Council for Harmonisation of Technical Requirements for Pharmaceuticals for Human Use) guidance, was considered a threshold of particular concern for degeneration to tachyarrhythmias and assessed for critical outcomes. Worst QTcF indicates the highest treatment-emergent averaged QTcF result (scheduled or unscheduled) observed centrally or locally. As moxifloxacin was replaced by levofloxacin in the control regimen during the study, comparisons of regimens control-mox/control-lev were conducted with participants in the oral regimen who were concurrently randomised with each subset of control regimen (control-mox/control-lev). All randomised participants received ≥1 dose of their assigned regimen and were included in the intention-to-treat (ITT) population.

Treatment-emergent adverse events (AEs), cardiovascular abnormalities observed from ECGs and reported as AEs (Medical Dictionary for Regulatory Activities [MedDRA] preferred term ‘electrocardiogram QT prolonged’), cardiac disorders based on the MedDRA System Organ Class of the same name, and QTcF abnormalities were assessed. ECG QT prolongation events were graded according to the Division of AIDS criteria. Relatedness to BDQ was assessed for serious AEs.^18,20^ An independent committee (two infectious disease physicians and a cardiologist) who were unaware of treatment group assignment reviewed all deaths and classified the probable cause of death as cardiac structural, cardiac arrhythmic (probable or possible sudden cardiac death), TB-related, or HIV-related (full results previously reported).^21^

Adjudication did not attribute causality to individual drugs; deaths were adjudicated by cause category (TB; HIV-related; cardiac; or other) rather than regimen-specific or drug-specific causality; as such, these classifications do not correspond numerically to investigator-reported AEs.

### Statistical analysis

Time to first QTcF ≥500 ms was analysed using the Kaplan–Meier product limit estimator with the log-rank test for estimating difference in median time to event between regimens, and Cox-proportional hazards model to determine the hazard ratio (HR) estimating the difference in risk between the two 40-week regimens (control vs. oral regimen). ECG results are presented for the treatment phase, whereas time to first QTcF and AEs related to cardiovascular disorders are reported for the whole study duration (treatment and/or follow-up phase). Categorical outlier analyses, including QTcF ≥500 ms and worst change from baseline ≥60 ms, were performed – these represent the established regulatory thresholds for assessment of proarrhythmic risk. Subgroup analyses of worst QTcF by sex, country, and HIV status were conducted; these analyses were descriptive in nature, with no formal analyses planned or performed, as this study was not powered for such comparison. These analyses are exploratory and should be interpreted cautiously.

### Ethical statement

The protocol, amendments, and other relevant documents were approved by the IRB/IEC before the study was initiated or amendments implemented. This study was conducted in accordance with the Declaration of Helsinki and was consistent with Good Clinical Practice and applicable regulatory requirements. Participants or their legally acceptable representatives provided their informed written consent to participate. Personal data from participants enrolled in this study were limited to those data necessary to investigate the efficacy, safety, quality, and utility of the investigational study intervention(s) used in this study and were collected and processed with adequate precautions to ensure confidentiality and compliance with applicable data privacy protection laws and regulations.

## RESULTS

During the study period of 27 March 2016 (first site opened) to 2 August 2020 (last observation from last participant), 588 participants were randomised (WHO: *n* = 32; control: *n* = 202 [control-mox: *n* = 140; control-lev: *n* = 62]; oral: *n* = 211; 6-month: *n* = 143). Baseline demographics and disease characteristics have been published.^18^ Of the 588 participants in the ITT population, 236 (40%) were female, and the median (range) age was 32.7 years (16–69). Participants were most commonly from India (*n* = 148, 25.2%) or Mongolia (*n* = 130, 22.1%), followed by South Africa (*n* = 92, 15.6%), Ethiopia (*n* = 67, 11.4%), Moldova (*n* = 63, n = 10.7%), Uganda (*n* = 56, 9.5%), and Georgia (*n* = 32, 5.4%). Ninety-seven (16.5%) participants were living with HIV, most of whom were from South Africa and Uganda; all received concomitant antiretroviral medication during the study. Randomisation to the WHO regimen was stopped early, and the data presented henceforth are for control, oral, and 6-month regimens only.

### ECG parameters over time

**Figure 1.**
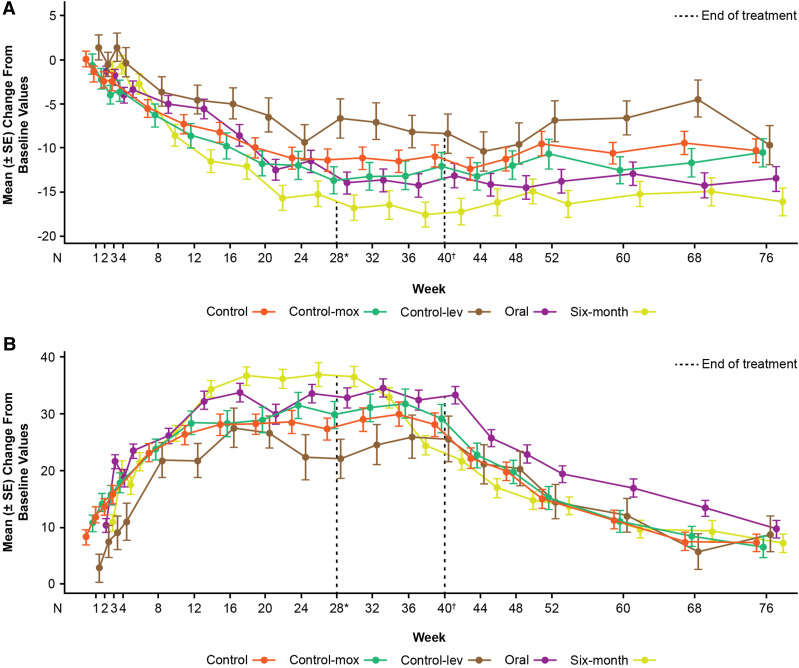
Change from baseline over time in **A:** heart rate (beats per minute) and **B:** QTcF (ms) (ITT population). Details of the regimens are shown in the Table. *End-of-treatment for 6-month regimen. ^†^End-of-treatment for 9-month (oral) regimen. Data represent the mean change (± SE) from baseline of the heart rate and QTcF data for participants based on observed values at each timepoint. ITT= intention-to-treat; lev = levofloxacin; mox = moxifloxacin; SE = standard error.

Overall, no sustained or clinically relevant changes from baseline were observed for mean PR duration, QRS duration, and RR duration for any of the regimens during the treatment phase (data not shown). A gradual decrease in heart rate from baseline to Week 24 during the treatment phase was observed for all regimens (Figure 1A). The smallest decrease was observed in control (11.2 bpm [control-mox: 12.0 bpm; control-lev: 9.4 bpm]) and oral (11.5 bpm) regimens, with the greatest decrease in the 6-month regimen (15.2 bpm) (Figure 1A).

Over the treatment and/or follow-up phase, 148 participants experienced ≥1 Grade ≥ 3 ECG QT prolongation (control: *n* = 50/202, 25%; oral: *n* = 59/211, 28%; 6-month: *n* = 39/143, 27%). Mean QTcF values gradually increased from baseline until a plateau was reached between Weeks 10 and 14, followed by a gradual decrease upon treatment completion (Figure 1B). The difference in mean QTcF change from baseline between the BDQ-containing regimens (oral or 6-month) and control was <10 ms at all timepoints throughout the treatment phase (Figure 1B).

**Figure 2.**
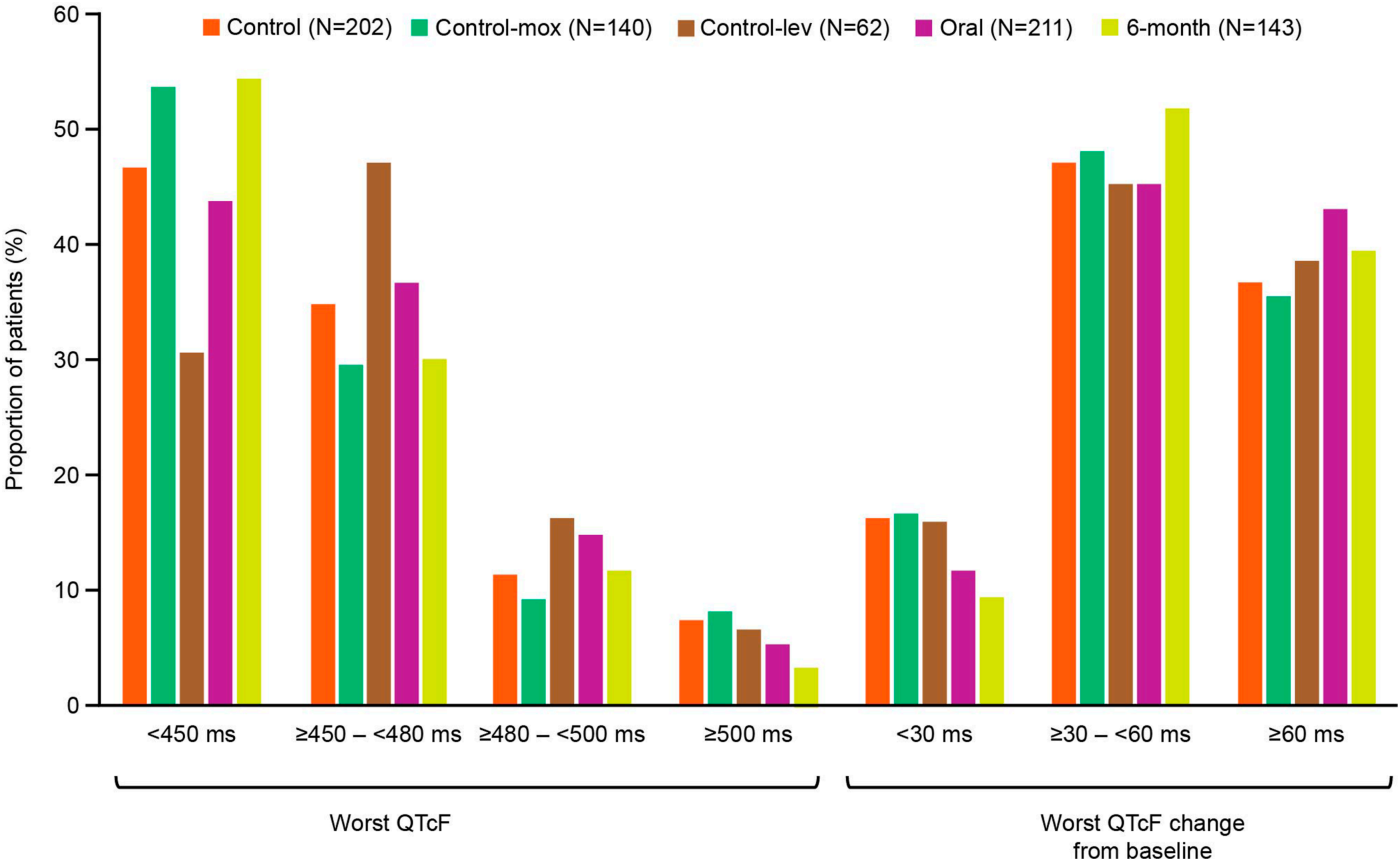
Worst QTcF in the treatment phase (ITT population). ITT = intention-to-treat; lev = levofloxacin; mox = moxifloxacin; QTcF = Fridericia’s corrected QT interval.

**Figure 3.**
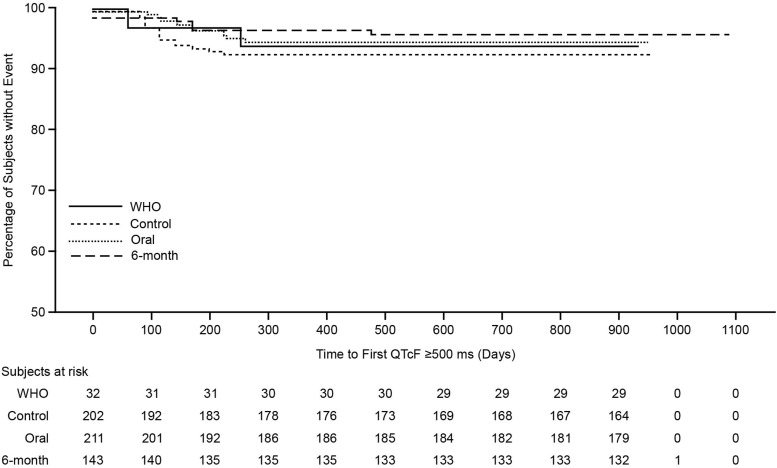
Kaplan–Meier figure showing time to first QTcF ≥500 ms (treatment and/or follow-up phase; ITT population). ITT = intention-to-treat; QTcF = Fridericia’s corrected QT interval; WHO = World Health Organization.

QTcF values ≥500 ms during the treatment phase were reported in 7.4% (*n* = 15 [control; control-mox: 7.9%, *n* = 11; control-lev: 6.5%, *n* = 4]), 5.2% (*n* = 11 [oral]), and 3.5% (*n* = 5 [6-month]) of participants (Supplementary Data Table S1; Figure 2). Throughout the treatment and/or follow-up phase, the risk of a participant experiencing ≥1 episode of QTcF ≥500 ms was consistent between control and oral regimens overall (unadjusted HR 1.43 [95% confidence interval (CI): 0.66, 3.11]; Supplementary Data Table S2; Figure 3). This finding was consistent in analyses restricted to control-lev versus concurrent oral (HR 0.85 [95% CI: 0.23, 3.17]) and control-mox versus concurrent oral (HR 1.89 [95% CI: 0.70, 5.12]; Supplementary Data Table S2).

During the treatment phase, the proportion of participants with the worst QTcF change from baseline of ≥30 to <60 ms was lowest in the oral regimen (45.0%, *n* = 95), but similar to that observed in the remaining regimens: control (47.0%, *n* = 95); control-mox (47.9%, *n* = 67); control-lev (45.2%, *n* = 28); and 6-month (51.7%, *n* = 74) (Supplementary Data Table S1; Figure 2). Similar trends were observed for worst QTcF change from baseline ≥60 ms (control: 36.6% [control-mox: 35.7%, *n* = 50; control-lev: 38.7%, *n* = 24], *n* = 74; oral: 43.1%, *n* = 91; 6-month: 39.2%, *n* = 56) (Supplementary Data Table S1; Figure 2).

### QTcF abnormalities by country, HIV status, and sex

The worst QTcF values (treatment and/or follow-up phase) by country are shown in Supplementary Data Table S3. When presented by country, the proportion of participants with worst QTcF values ≥ 500 ms was highest in Mongolia (control: 26.1%; control-mox: 33.3%; control-lev: 18.2%; oral: 14.6%; 6-month: 12.0%) compared with other countries, ranging from 2.1% to 12.5% across all other countries (Supplementary Data Table S3).

By HIV status, there was no signal for an increased risk of QT prolongation based on the worst QTcF values and worst change from baseline QTcF (Supplementary Data Table S4 and S5). However, any observations should be interpreted with caution due to the low sample size for participants living with HIV (*N* = 97). Of these 97 participants randomised to BDQ-containing oral and 6-month, one participant (6-month regimen) had a QTcF value ≥500 ms during the treatment phase.

Analysis by sex showed that the proportion of participants with worst QTcF ≥500 ms during the treatment phase was numerically slightly higher in women (control: 9.9%, *n* = 8 [control-mox: 8.8%, *n* = 5; control-lev: 12.5%, *n* = 3]; oral: 6.3%, *n* = 5; 6-month: 1.7%, *n* = 1) than men (control: 5.8%, *n* = 7 [control-mox: 7.2%, *n* = 6; control-lev: 2.6%, *n* = 1]; oral: 4.5%, *n* = 6; 6-month: 6.0%, *n* = 5) for all regimens, except 6-month.

### Other cardiac-related safety

In terms of other cardiac-related AEs over the treatment and/or follow-up phase, three participants were reported by investigators to have had at least one Grade ≥3 cardiac disorder: one participant (6-month regimen) had an arrhythmia, one participant (oral regimen) had congestive cardiac failure, and one participant had an acute coronary syndrome (oral regimen). The acute coronary syndrome case resulted in death (Grade 5) and was assessed unrelated to BDQ by the investigator. The highest measured QTcF during treatment and follow-up was <460 ms in all three participants with a Grade ≥3 cardiac disorder.

In total, 21 (3.8%) participants died (control: *n* = 8 [4.0%]; oral: *n* = 11 [5.2%]; 6-month: *n* = 2 [1.4%]); based on an independent cause-of-death adjudication, six deaths were attributed to cardiac-related causes, four deaths were assessed as due to arrhythmic causes, with no evidence of QT prolongation: possible sudden cardiac death (one in control and three in oral regimen), and a further two deaths were assessed as due to structural cardiac causes (control: *n* = 1; oral: *n* = 1). The measured QTcF was <460 ms in all deceased participants, and no cases of Torsades de Pointes were reported.

## DISCUSSION

In the STREAM Stage 2 trial, QTcF interval changes remained stable over a treatment duration of up to 40 weeks across BDQ-containing regimens – both oral and 6-month – and the BDQ-free control. All regimens included concomitant administration of clofazimine and fluoroquinolones, which are known to potentially prolong QT intervals. Importantly, the differences in mean QTcF change from baseline between each BDQ-containing regimen and the control consistently remained within 10 ms throughout treatment; QTcF intervals initially increased during the early phase, plateaued during ongoing treatment, and gradually declined after treatment cessation – similar to the trend observed in the control group. Heart rate trends displayed an inverse pattern, decreasing initially and stabilising, mirroring QTcF changes. Cardiac AEs were infrequent, and no cases of Torsades de Pointes were reported. Given the prominence of BDQ in MDR-TB treatment regimens currently, we believe the data that provide additional information about the specific contribution of BDQ to QT prolongation are clinically meaningful.

Previous research suggests that the QT-prolonging effect of BDQ is primarily driven by its major metabolite, M2, and that the maximal QTcF prolongation associated with BDQ occurs at peak plasma concentration, typically at the end of the 2-week loading phase and decreasing over time.^9^ In this trial, the greatest QTcF elevations occurred within the first 6–8 weeks, despite declining M2 plasma levels post-loading, likely due to interaction effects with other QT-prolonging drugs, with a maximum QTcF increase of approximately 10 ms. The potential for a synergistic effect of BDQ when used in combination with other QT-prolonging agents (such as clofazimine and fluoroquinolones) was not formally assessed, but the cumulative effect does not appear to be more than additive, consistent with prior pharmacokinetic and pharmacodynamic data.^9,22,23^ Importantly, QTcF prolongations reverted after treatment ended, indicating that the effects are reversible.^10,24^

The observed relationship between heart rate and QTcF underscores the influence of clinical improvement in TB disease on cardiac parameters – higher baseline heart rates tend to decrease over the course of effective treatment, influencing QTcF measurements.^25^ Thus, the changes from baseline in QTcF reported in this analysis are likely to be biased upward by the decrease in heart rate over time. The influence of heart rate variability has been recognised as one of the shortcomings of QTcF measurements. Analyses applying exposure-QT modelling to separate the HR effect from the drug effect have demonstrated that the contribution of BDQ to QT prolongation remains modest with a mean QTc increase of 7.9 ms at end of 2-week loading period.^26,27^ Time-varying QTc methods, accounting for the time-dependent HR, were also developed and validated for BDQ, showing a similarly modest mean QT increase of 7 ms at the end of the 2-week loading.^26–28^

The subgroup analyses suggest that susceptibility to QTc prolongation may vary across patient-level and population-level characteristics. In particular, the numerically higher proportion of QTcF ≥500 ms observed in women is consistent with the well-described predisposition of women to QT prolongation.^29^ There were also geographical variations noted, with Mongolia showing a higher proportion of individuals with worst QTcF ≥500 ms.^30^ Prior studies have reported regional associations with QT prolongation,^29,30^ as well as described heritable contributions to QTc variability in Mongolian populations.^31^ HIV status was not found to be associated with QTcF prolongation in this analysis, although prior studies^16,32–34^ have demonstrated higher predisposition to QT prolongation related to advanced disease states and drug–drug interactions, among other factors; however, given the small sample sizes, cautious interpretation is warranted. These data highlight the need to consider demographic factors in consideration of QT prolongation.

Overall, there was a low incidence of cardiac AEs, highlighting that the increased QTcF interval, which resolved over the course of the study, did not result in a concerning frequency of clinically relevant cardiac-related AEs in either BDQ-containing regimens or the BDQ-free control regimen.

This study’s prospective, longitudinal randomised design and comprehensive ECG monitoring allowed for detailed analysis of QTcF prolongation and its clinical implications. Additionally, the diverse study population, encompassing a wide range of participants across different geographical regions, age groups, and sexes, enhances the generalisability of the results. However, several limitations merit consideration. Limitations of the study include the small sample size for HIV-positive (n = 97) and the 6-month regimen (n = 143) participants; thus, any observed differences should be interpreted with caution. Of note, QT management during the trial aimed to maintain QTcF values below 500 ms, potentially leading to treatment modifications that could influence outcomes. Nonetheless, these findings are consistent with recent findings from pragmatic studies evaluating short-course BDQ-based regimens for rifampicin-resistant TB, which highlight that clinically significant QTc prolongation is rare with close monitoring.^35^ This analysis extends the primary STREAM Stage 2 safety reporting by drawing on more detailed, longitudinal ECG data to characterise the temporal evolution of QTc changes during and after treatment.

## CONCLUSION

These data reinforce existing evidence from clinical trials and observational studies regarding the cardiac safety profile of MDR-TB treatment regimens and indicate that the addition of BDQ to treatment regimens is associated with a mean QTcF prolongation of <10 ms. The effect is generally additive in the presence of other QT-prolonging drugs and rarely linked to significant cardiac events when supported by careful monitoring and appropriate treatment modifications. Given the extensive global adoption of BDQ in MDR-TB management, understanding its QT-prolonging profile can assist clinicians in designing safe and effective regimens, with appropriate risk mitigation.

## Supplementary Material
