# Supplementary material for: QT prolongation in participants receiving bedaquiline-containing regimens: analysis of data from Phase 3 STREAM Stage 2
Source: IJTLD Open. 2026 May 11;3(5):312–9. doi: 10.5588/ijtldopen.25.0652 (PMC13160264; doi:10.5588/ijtldopen.25.0652)
Supplement: Supplementary file 1 [file ijtldopen25-0652_supplementarydata1.pdf]

Supplementary Tables

Supplementary Table 1. Worst QTcF (values and change from baseline; ms) in the treatment phase (ITT population)

	Regimen				
	Control (*N*=202)	Control-mox (*N*=140)	Control-lev (*N*=62)	Oral (*N*=211)	6-month (*N*=143)
Worst QTcF, *n* (%)					
<450 ms	94 (46.5)	75 (53.6)	19 (30.6)	92 (43.6)	78 (54.5)
≥450 – <480 ms	70 (34.7)	41 (29.3)	29 (46.8)	77 (36.5)	43 (30.1)
≥480 – <500 ms	23 (11.4)	13 (9.3)	10 (16.1)	31 (14.7)	17 (11.9)
≥500 ms	15 (7.4)	11 (7.9)	4 (6.5)	11 (5.2)	5 (3.5)
Worst QTcF change from baseline, *n* (%)					
<30 ms	33 (16.3)	23 (16.4)	10 (16.1)	25 (11.8)	13 (9.1)
≥30 – <60 ms	95 (47.0)	67 (47.9)	28 (45.2)	95 (45.0)	74 (51.7)
≥60 ms	74 (36.6)	50 (35.7)	24 (38.7)	91 (43.1)	56 (39.2)

Details of the regimens are shown in Table 1.

Worst QTcF was defined as the highest post-baseline averaged ECG value (scheduled or unscheduled, centrally or locally measured) emerging during the treatment phase.

ECG electrocardiogram; ITT, intention-to-treat; lev, levofloxacin; mox, moxifloxacin; QTcF, QT corrected by Fridericia's formula.

Supplementary Table 2. Kaplan-Meier and Cox regression analysis of time to first QTcF ≥500 ms (treatment and/or follow-up phase; ITT population)

Time to first QTcF ≥500 ms	Regimen (overall)		Regimen			
	Control (*N*=202)	Oral (*N*=211)	Control-mox (*N*=140)	Concurrent oral (*N*=144)	Control-lev (*N*=62)	Concurrent oral (*N*=67)
Number censored, *n* (%)	187 (92.6)	200 (94.8)	129 (92.1)	138 (95.8)	58 (93.5)	62 (92.5)
Number with event[¶], *n* (%)	15 (7.4)	11 (5.2)	11 (7.9)	6 (4.2)	4 (6.5)	5 (7.5)
	Pairwise comparisons*					
	-	Control vs oral	-	Control-mox vs oral	-	Control-lev vs oral
Log-rank *P*-value	-	0.368	-	0.200	-	0.809
Proportional hazards model						
HR	-	1.43	-	1.89	-	0.85
95% CI	-	0.66, 3.11	-	0.70, 5.12	-	0.23, 3.17
P-value	-	0.370	-	0.208	-	0.809

Details of the regimens are shown in Table 1.

*Pairwise comparisons were based on concurrently randomized participants. event' ¶An event was defined as a QTcF ≥500 ms

CI, confidence interval; HR, hazard ratio; ITT, intention-to-treat; lev, levofloxacin; mox, moxifloxacin; QTcF, QT corrected by Fridericia's formula.

Supplementary Table 3. Worst QTcF (values; ms) by country (ITT population; treatment and/or follow-up phase)

Worst QTcF, *n* (%)	Regimen				
	Control (*N*=202)	Control-mox (*N*=140)	Control-lev (*N*=62)	Oral (*N*=211)	6-month (*N*=143)
Ethiopia, *n*	21	19	2	20	20
<450 ms	16 (76.2)	15 (78.9)	1 (50.0)	16 (80.0)	16 (80.0)
≥450 – <480 ms	5 (23.8)	4 (21.1)	1 (50.0)	3 (15.0)	4 (20.0)
≥480 – <500 ms	0	0	0	0	0
≥500 ms	0	0	0	1 (5.0)	0
Georgia, *n*	13	7	6	12	7
<450 ms	5 (38.5)	2 (28.6)	3 (50.0)	7 (58.3)	3 (42.9)
≥450 – <480 ms	4 (30.8)	2 (28.6)	2 (33.3)	4 (33.3)	2 (28.6)
≥480 – <500 ms	3 (30.8)	3 (42.9)	1 (16.7)	1 (8.3)	2 (28.6)
≥500 ms	0	0	0	0	0
India, *n*	47	47	0	48	48
<450 ms	33 (70.2)	33 (70.2)	0	28 (58.3)	34 (70.8)
≥450 – <480 ms	11 (23.4)	11 (23.4)	0	14 (29.2)	11 (22.9)
≥480 – <500 ms	2 (4.3)	2 (4.3)	0	6 (12.5)	2 (4.2)
≥500 ms	1 (2.1)	1 (2.1)	0	0	1 (2.1)
Moldova, *n*	25	8	17	26	8
<450 ms	9 (36.0)	4 (50.0)	5 (29.4)	6 (23.1)	1 (12.5)
≥450 – <480 ms	13 (52.0)	3 (37.5)	10 (58.8)	12 (46.2)	2 (25.0)
≥480 – <500 ms	3 (12.0)	1 (12.5)	2 (11.8)	5 (19.2)	4 (50.0)
≥500 ms	0	0	0	3 (11.5)	1 (12.5)
Mongolia, *n*	46	24	22	48	25
<450 ms	6 (13.0)	3 (12.5)	3 (13.6)	6 (12.5)	5 (20.0)
≥450 – <480 ms	18 (39.1)	10 (41.7)	8 (36.4)	21 (43.8)	11 (44.0)

≥480 – <500 ms	10 (21.7)	3 (12.5)	7 (31.8)	14 (29.2)	6 (24.0)
≥500 ms	12 (26.1)	8 (33.3)	4 (18.2)	7 (14.6)	3 (12.0)
South Africa, *n*	28	28	0	32	26
<450 ms	15 (53.6)	15 (53.6)	0	15 (46.9)	13 (50.0)
≥450 – <480 ms	9 (32.1)	9 (32.1)	0	14 (43.8)	10 (38.5)
≥480 – <500 ms	2 (7.1)	2 (7.1)	0	3 (9.4)	2 (7.7)
≥500 ms	2 (7.1)	2 (7.1)	0	0	1 (3.8)
Uganda, *n*	22	7	15	25	9
<450 ms	8 (36.4)	1 (14.3)	7 (46.7)	11 (44.0)	4 (44.4)
≥450 – <480 ms	10 (45.5)	4 (57.1)	6 (40.0)	11 (44.0)	3 (33.3)
≥480 – <500 ms	4 (18.2)	2 (28.6)	2 (13.3)	3 (12.0)	2 (22.2)
≥500 ms	0	0	0	0	0

Details of the regimens are shown in Table 1.

ITT, intention-to-treat; lev, levofloxacin; mox, moxifloxacin.

Supplementary Table 4. Worst QTcF (values; ms) by HIV status (ITT population; treatment phase)

Worst QTcF, *n* (%)	Regimen				
	Control (*N*=202)	Control-mox (*N*=140)	Control-lev (*N*=62)	Oral (*N*=211)	6-month (*N*=143)
HIV-positive (CD4 count <350 cells/mm^3)					
Baseline, *n*	16	14	2	18	12
<450 ms	15 (94)	13 (93)	2 (100)	18 (100)	12 (100)
≥450 – <480 ms	1 (6)	1 (7)	0	0	0
≥480 – <500 ms	0	0	0	0	0
≥500 ms	0	0	0	0	0
Treatment phase, *n*	16	14	2	18	12
<450 ms	8 (50)	6 (43)	2 (100)	9 (50)	9 (75)
≥450 – <480 ms	6 (38)	6 (43)	0	7 (39)	3 (25)
≥480 – <500 ms	2 (13)	2 (14)	0	2 (11)	0
≥500 ms	0	0	0	0	0
HIV-positive (CD4 count ≥350 cells/mm^3)					
Baseline, *n*	16	14	2	18	12
<450 ms	16 (100)	14 (100)	2 (100)	18 (100)	12 (100)

≥450 – <480 ms	0	0	0	0	0
≥480 – <500 ms	0	0	0	0	0
≥500 ms	0	0	0	0	0
Treatment phase, *n*	15	13	2	16	14
<450 ms	9 (60)	8 (62)	1 (50)	10 (63)	7 (50)
≥450 – <480 ms	4 (27)	4 (31)	0	6 (38)	4 (29)
≥480 – <500 ms	2 (13)	2 (13)	1 (50)	0	2 (14)
≥500 ms	0	0	0	0	1 (7)
HIV-negative					
Baseline, *n*	171	113	58	177	117
<450 ms	170 (99)	113 (100)	57 (98)	177 (100)	117 (100)
≥450 – <480 ms	1 (1)	0	1 (2)	0	0
≥480 – <500 ms	0	0	0	0	0
≥500 ms	0	0	0	0	0
Treatment phase, *n*	171	113	58	177	117
<450 ms	77 (45)	61 (54)	16 (28)	73 (41)	62 (53)
≥450 – <480 ms	60 (35)	31 (27)	29 (50)	64 (36)	36 (31)

≥480 – <500 ms	19 (11)	10 (9)	9 (16)	29 (16)	15 (13)
≥500 ms	15 (9)	11 (10)	4 (7)	11 (6)	4 (3)

Details of the regimens are shown in Table 1.

HIV, human immunodeficiency virus, ITT, intention-to-treat; lev, levofloxacin; mox, moxifloxacin.

Supplementary Table 5. Worst QTcF change from baseline (ms) by HIV status (ITT population; treatment phase)

	Regimen				
Worst QTcF, *n* (%)	Control (*N*=202)	Control-mox (*N*=140)	Control-lev (*N*=62)	Oral (*N*=211)	6-month (*N*=143)
HIV-positive (CD4 count <350 cells/mm^3)					
Treatment phase, *n*	16	14	2	18	12
<30 ms	5 (31.3)	4 (28.6)	1 (50.0)	1 (5.6)	3 (25.0)
≥30 – <60 ms	7 (43.8)	7 (50.0)	0	11 (61.1)	3 (25.0)
≥60 ms	4 (25.0)	3 (21.4)	1 (50.0)	6 (33.3)	6 (50.0)
HIV-positive (CD4 count ≥350 cells/mm^3)					
Treatment phase, *n*	15	13	2	16	14
<30 ms	5 (33.3)	5 (38.5)	0	3 (18.8)	1 (7.1)
≥30 – <60 ms	6 (40.0)	5 (38.5)	1 (50.0)	7 (43.8)	9 (64.3)
≥60 ms	4 (26.7)	3 (23.1)	1 (50.0)	6 (37.5)	4 (28.6)
Follow-up, *n*	10	8	2	15	14
<30 ms	5 (50.0)	5 (62.5)	0	5 (33.3)	4 (28.6)
≥30 – <60 ms	4 (40.0)	2 (25.0)	2 (100.0)	8 (53.3)	7 (50.0)
≥60 ms	1 (10.0)	1 (12.5)	0	2 (13.3)	3 (21.4)
HIV-negative					

Treatment phase, *n*	171	113	58	177	117
<30 ms	23 (14)	14 (12)	9 (16)	21 (12)	9 (8)
≥30 – <60 ms	82 (48)	55 (49)	27 (47)	77 (44)	62 (53)
≥60 ms	66 (39)	44 (39)	22 (38)	79 (45)	46 (39)

Details of the regimens are shown in Table 1.

HIV, human immunodeficiency virus, ITT, intention-to-treat; lev, levofloxacin; mox, moxifloxacin.
